# The Triple Flexion Response in Friedrich’s Ataxia

**DOI:** 10.7759/cureus.96080

**Published:** 2025-11-04

**Authors:** Alvee Saluja, Akhil Sahib, Vikas Yadav

**Affiliations:** 1 Neurology, Lady Hardinge Medical College, New Delhi, IND; 2 Neurology, Lady Hardinge Medical College and Associated Hospitals, New Delhi, IND; 3 Radiology, Lady Hardinge Medical College and Associated Hospitals, New Delhi, IND

**Keywords:** corticospinal tracts, dorsal reticulospinal tracts, frataxin, friedrich's ataxia, triple flexion response

## Abstract

Friedrich’s ataxia is the most common inherited ataxia and predominantly affects the dorsal root ganglia, sensory peripheral nerves, posterior columns, the corticospinal and spinocerebellar tracts. A 19-year-old male presented with an insidious onset, gradually progressive gait ataxia and bilateral foot drop for the past 10 years, and slurring of speech for the past 5 years. The neurological examination revealed dorsal spine scoliosis, bilateral foot drop (power of grade 1/5 at the bilateral ankles), diminished to absent deep tendon reflexes, and impaired finger-nose-finger testing. The joint position and vibration sense were absent till the bilateral knees. Plantar reflex testing revealed extension of the great toe with dorsiflexion of the ankle, knee, and hip (the triple flexion response). The nerve conduction studies revealed absent sensory nerve action potentials. Magnetic resonance imaging (MRI) demonstrated mild cerebellar atrophy and atrophy of the cervical spinal cord and medulla. Frataxin gene analysis revealed >66 homozygous GAA repeats (reference 5-33 repeats), confirming Friedrich’s ataxia. The triple flexion response consists of simultaneous ankle, hip, and knee flexion when stimulating the sole or even spontaneously. Unlike the normal withdrawal response, the triple flexion response is stereotyped, consistent, and reproducible despite multiple stimulations. The descending corticospinal and dorsal (particularly the medullary/medial) reticulospinal tract degeneration in Friedrich’s ataxia may cause disinhibition of spinal interneurons involved in the lower limb flexion reflex arc, producing the triple flexion response. Thus, the triple flexion response is a significant diagnostic clue highlighting the marked corticospinal tract involvement in advanced Friedrich's ataxia.

## Introduction

Friedrich’s ataxia is the most common inherited ataxia with an estimated worldwide prevalence of 0.5 cases per 100,000 individuals [1]. This autosomal recessive disorder results from the pathogenic homozygous GAA trinucleotide repeat expansion in intron 1 of the Frataxin (FXN) gene on chromosome 9q21 [2]. Impaired FXN gene functioning leads to a deficiency of the frataxin protein, which plays a key role in iron homeostasis, cellular redox reaction, mitochondrial ATP production, and cellular oxidative stress response [3,4]. Friedrich’s ataxia predominantly affects the dorsal root ganglia, sensory peripheral nerves, the posterior columns, corticospinal tracts, and the dentate nucleus of the cerebellum, along with the spinocerebellar tracts [5,6]. The triple flexion response consists of stereotyped ankle, knee, and hip flexion on cutaneous stimulation (or spontaneously), signifying reflex spinal automatism [7]. The reflex is produced when lesions of the descending upper motor neuron tracts cause release of the spinal motor neurons from cortical inhibitory influence. In contrast to the Babinski’s sign (that involves great toe extension, fanning of the other toes, and is limited to the foot), the triple flexion response reflects spread of the reflex arc to the L2 and L3 myotomes, resulting in concomitant hip and knee flexion, and indicates profound corticospinal tract dysfunction [8]. Although the reflex has been previously described, it has seldom been video recorded in published literature. Furthermore, the triple flexion response has not been previously reported among patients with Friedrich’s ataxia. This case highlights this rare but important clinical finding in Friedrich’s ataxia and further discusses the need to correctly identify this sign of corticospinal tract dysfunction in clinical practice.

## Case presentation

A 19-year-old, right-handed male born of a consanguineous marriage via a full-term normal vaginal delivery had normal milestones. At nine years of age, he started having recurrent falls due to the sudden inward twisting of the ankle. Two years afterward, he developed progressive imbalance while walking and bilateral foot drop. He also noted that the imbalance in walking was worse with the eyes closed (particularly when washing his face) and in dimly lit surroundings. There was progressive incoordination in reaching out towards objects (such as a glass of water), while brushing his teeth, and while walking. Four years after symptom onset, he started having difficulty getting up from a sitting position. There has been a slurring of speech for the past 5 years. He was confined to a wheelchair since the age of 15 years. There was no history suggestive of cognitive decline, limb stiffness, sensory loss, diminution of vision, extraocular movement abnormality, hearing loss, swallowing difficulty, or abnormal involuntary body movements. There was no history of chest pain, palpitation, shortness of breath, or orthopnea. His elder sister had similar symptoms and had expired at the age of 14 years.

Examination revealed an asthenic build. Higher mental functions were normal, and the Mini-Mental Status examination was 28/28 (the patient could not perform the writing task and figure copying). Wasting of the thenar and hypothenar eminences, bilateral calves, peronei group, and small muscles of the feet was noted. There was scoliosis at the level of the mid-dorsal spine with secondary curves in the cervical and lumbar regions. The motor system examination revealed gross hypotonia in all four limbs. Power at the shoulder, elbow, and wrist joints in all ranges of motion was 5/5 by the modified research council (MRC) grading system [9]. Power at the hip joint was MRC grade 1/5. Power at the knee joint in flexion and extension was 1/5 and 2/5, respectively. There was bilateral foot drop (power MRC grade 1/5 at the bilateral ankles). Crude touch, pain, and temperature sensations were preserved in all four limbs. However, joint position and vibration sense were absent till the knees. All the deep tendon reflexes were diminished to absent (Video 1).

**Video 1:** Diminished to absent deep tendon jerks in upper and lower limbs The video highlights absent supinator reflex and diminished biceps and triceps reflexes in the upper limbs. The bilateral knee and ankle jerks are absent as well.

There was dysdiadokokinesia (inability to perform rapid alternating movements such as supination and pronation) and impaired finger-nose-finger testing on cerebellar testing (Video 2).

**Video 2:** Impaired finger-nose-finger testing and dysdiadokokinesia in the upper limbs The video highlights incoordination and impaired finger-nose-finger testing in the bilateral upper limbs. Bilateral dysdiadokokinesia is noted in the upper limbs as well.

Romberg’s sign could not be assessed as the patient was bed-bound. Plantar reflex testing revealed extension of the great toe with dorsiflexion of the ankle, knee, and hip that was stereotyped (repetitive) and reproducible despite multiple stimulations (the triple flexion response) (Video 3).

**Video 3:** The triple flexion response The video highlights the simultaneous ankle dorsiflexion (with great toe extension), knee, and hip flexion (triple flexion) on stroking the sole. Note the upgoing (dorsiflexion) of the great toe and ankle, followed by the rapid flexion of the knee and hip. Since there is simultaneous flexion of the ankle, knee, and hip, the response is denoted as "triple flexion." A stereotyped (repetitive) and reproducible response is noted on repeated stimulation.

Apart from low vitamin B12 and vitamin D3 levels, all other routine blood investigations were within normal limits (Table 1).

**Table 1:** Blood investigations of the patient g/dL- grams/deciliter; cells/mm3- cell per cubic ml; %- percentage; mg/dL- milligrams/deciliter; IU/L-International Units/Liter; mEq/L- milliequivalents/Liter; micro IU/ml- micro International units/milliliter; ng/ml- nanograms/milliliter; pg/ml- picograms/milliliter

S.No.	Parameter (units)	Value	Reference value
1.	Hemoglobin (g/dL)	15.1	13-18
2.	Total leukocyte count (cells/mm^3^)	5080	4000-11000
3.	Platelet counts (cells/mm^3^)	163000	150000-450000
4.	RBC counts (cells/mm^3^)	5.03 million	4.5-6 million
5.	Hematocrit (%)	44.1	40-54
6.	Total bilirubin (mg/dL)	1.34	0.6-1.3
7.	Aspartate aminotransferase (IU/L)	27	5-45
8.	Alanine aminotransferase (IU/L)	15	5-35
9.	Alkaline phosphatase (IU/L)	94	48-128
10.	Serum albumin (g/dL)	4.44	3.5-5.2
11.	Sodium (mEq/L)	141	136-145
12.	Potassium (mEq/L)	3.84	3.5-5.1
13.	Total calcium (mEq/L)	9.9	8.6-10.2
14.	Urea (mg/dL)	31	15-40
15.	Creatinine (mg/dL)	1.16	0.6-1.3
16.	Fasting blood sugar (mg/dL)	82	70-100
17.	Thyroid-stimulating hormone (micro IU/ml)	1.39	0.34-5.6
18.	Vitamin B12 (pg/ml)	136	180-911
19.	Vitamin D3 (ng/ml)	18.6	30-100
20.	Human immunodeficiency virus serology	Negative	Negative
21.	Hepatitis-B surface antigen serology	Negative	Negative
22.	Anti-hepatitis C serology	Negative	Negative
23.	Creatinine kinase (IU/L)	154.2	<171
24.	Anti-nuclear antibody	Negative	Negative
25.	Rheumatoid factor	Negative	Negative

**Figure 1:**
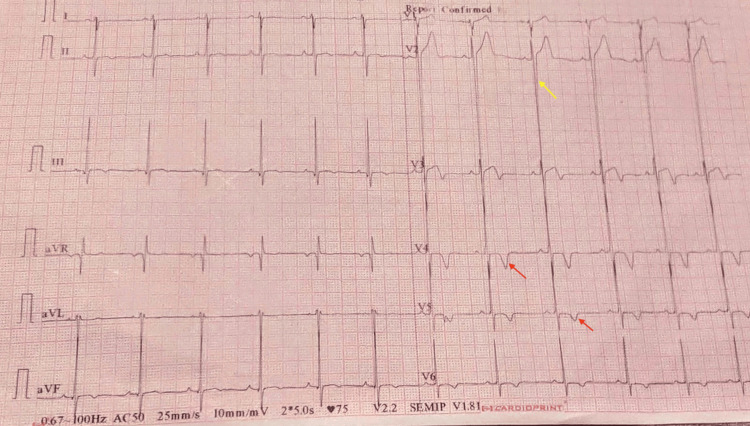
Electrocardiogram (ECG) of the patient The electrocardiogram of the patient (10 mm = 1 mV, speed-25mm/second) shows a deep S wave in the V2 precordial lead measuring 45 mm (yellow arrow) along with tall R waves in the left precordial leads (V4, V5, V6). The combined amplitude of the S wave in V2 and the R wave in V5 measures 65 mm (suggestive of left ventricular hypertrophy). ST-segment depression and T wave inversions (red arrows) are noted in the V4, V5, and V6 (left precordial leads), suggestive of the LV strain pattern.

The electrocardiogram was suggestive of a left ventricular strain pattern (Figure 1).

**Figure 2:**
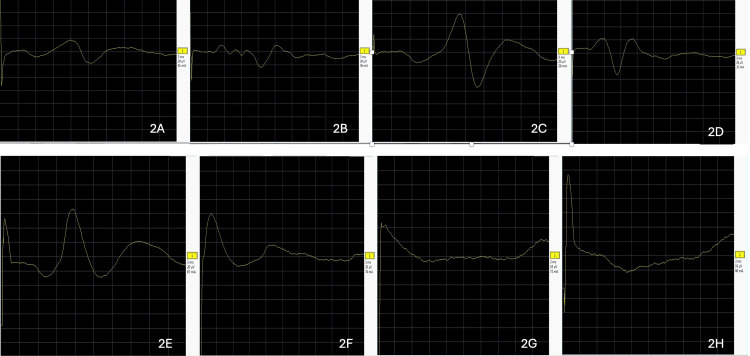
Sensory nerve conduction study graphs of the patient The sensory nerve conduction study shows absent sensory nerve action potentials (SNAPs) in the right median (2A), right ulnar (2B), left median (2C), left ulnar (2D), right sural (2E), left sural (2F), right superficial peroneal nerve (2G), and the left superficial peroneal nerves (2H) (sensitivity: 10-20 microvolt/division, sweep speed: 2-3 ms/division).

His 2D-echocardiography revealed hypertrophic cardiomyopathy. The nerve conduction studies revealed absent sensory nerve action potentials, along with mildly prolonged motor distal latencies and decreased motor conduction velocities, in all four limbs (Figures 2A-2H and Table 2).

**Table 2:** Sensory nerve conduction study values in our case and normal reference values ms- milliseconds, SNAP- sensory nerve action potential, m/s- meters/second, NR- not recordable The normal peak latency values should be less than or equal (≤) to the mentioned limits. Normal SNAP amplitude and conduction velocity values should be more than or equal (≥) to the mentioned limits.

Sensory nerve studied	Peak latency (ms)		SNAP amplitude (microvolts)		Conduction velocity (m/s)	
	Patient value	Normal reference value limit	Patient value	Normal reference value limit	Patient value	Normal reference value limit
Right median	NR	≤3.5	NR	≥5.0	NR	≥50
Right ulnar	NR	≤3.2	NR	≥5.0	NR	≥51
Left median	NR	≤3.5	NR	≥5.0	NR	≥50
Left ulnar	NR	≤3.2	NR	≥5.0	NR	≥51
Right sural	NR	≤4.4	NR	≥5.0	NR	≥40
Left sural	NR	≤4.4	NR	≥5.0	NR	≥40
Right superficial peroneal	NR	≤2.3	NR	≥4.7	NR	≥40
Left superficial peroneal nerve	NR	≤2.3	NR	≥4.7	NR	≥40

**Figure 3:**
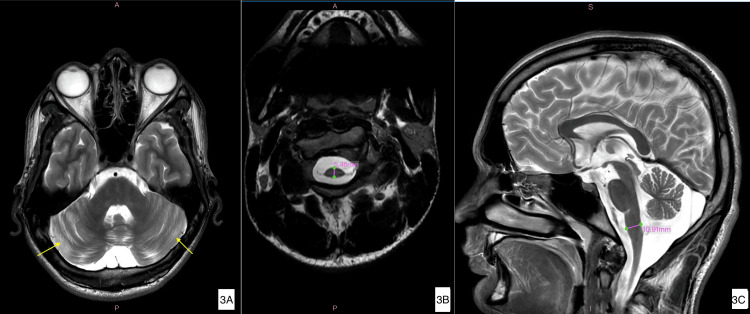
Magnetic resonance imaging (MRI) of the spinal cord and brain 3A. T2-weighted axial MRI of the brain showing prominent cerebellar folia (yellow arrows) suggestive of mild cerebellar atrophy. 3B. T2-weighted axial MRI of the cervical spinal cord (C3 level) showing reduced anteroposterior diameter of 5.46 mm, suggestive of cervical cord atrophy. (measuring marker (purple color); normal reference 8.0 mm). 3C. T2-weighted sagittal MRI brain highlighting mild medullary atrophy with an antero-posterior diameter of 10.91 mm (measuring marker (purple color); normal reference 13 mm)

Magnetic resonance imaging (MRI) of the brain and spinal cord demonstrated prominence of cerebellar folia (suggestive of mild cerebellar atrophy), and reduced anteroposterior diameter of the cervical spinal cord (measuring 5.46 millimetres, normal reference: 8 millimetres) and the medulla (measuring 10.91 millimetres, normal reference: 13 millimetres), suggestive of cervical spinal cord and medullary atrophy in our patient (Figure 3A-3C).

In view of an autosomal recessive inheritance pattern, adolescent-onset progressive cerebellar ataxia and dysarthria, large fibre sensory impairment, absent SNAP potentials, absent DTRs with extensor plantar response (and triple flexion response), and cervical spinal cord and medullary atrophy on MRI, Friedrich’s ataxia was considered the most likely diagnosis. The typical findings in Friedrich's ataxia, along with their clinical localization, are highlighted in Table 3.

**Table 3:** Summary of clinical, radiological, nerve conduction, and genetic features of Friedrich’s ataxia and their clinical localization

Finding	Finding in Friedrich’s Ataxia	Clinical localization
1. Motor examination	Distal and proximal weakness in the lower and upper limbs	Peripheral nerves, corticospinal tract degeneration
2. Sensory examination	Loss of joint position and vibration sense in the limbs (especially the lower limbs)	Dorsal root ganglia, posterior column, large fiber sensory nerves
3. Cerebellar examination	Impaired finger-nose finger testing, dysdiadokokinesia (inability to perform rapid alternating movement), scanning speech	Cerebellum and its connections (spinocerebellar tracts)
4. Gait	Ataxic gait	Dorsal root ganglia, Posterior column, large fiber sensory nerves, cerebellum and its connection
5. Scoliosis	Commonly present	Musculoskeletal deformity due to weakness
6. Deep tendon reflexes	Absent	Dorsal root ganglia, posterior column, large fiber sensory nerves
7. Plantar reflex	Bilateral extensor	Corticospinal tract degeneration
8. Triple flexion response	Not previously described	Corticospinal and medial/medullary reticulospinal tract degeneration
9. Electrocardiogram	Left ventricular hypertrophy, left ventricular strain	Cardiac muscle involvement
10. 2D-Echocardiography	Left ventricular hypertrophy, hypertrophic cardiomyopathy	Cardiac muscle involvement
11. Radiology	Cervical spinal cord and medullary atrophy, mild cerebellar atrophy	Corticospinal tract degeneration, dorsal root ganglia and posterior column degeneration, cerebellum and its connection
12. Nerve conduction studies	Absent sensory nerve action potentials	Dorsal root ganglia, posterior column, large fiber sensory nerves
13. Genetic testing	Homozygous pathogenic triplet repeat (GAA) expansion in the Frataxin gene	-----

Frataxin gene analysis revealed >66 homozygous GAA repeats (reference 5-33 repeats), suggestive of a full pathogenic allele mutation, confirming the diagnosis of Friedrich’s ataxia. The patient was advised physiotherapy exercises, oral idebenone (at 5 mg/kg body weight), oral coenzyme Q10 (400 mg/day), vitamin E (200 mg/day), levocarnitine (150 mg/day), high-dose vitamin B12(1500 micrograms/day), and oral calcium and vitamin D (500 mg/400 IU twice a day) supplementation. The family and the patient were counseled regarding the nature of the disease. He is currently under follow-up.

## Discussion

Friedrich’s ataxia is a neurodegenerative disorder resulting from the deficiency of the frataxin protein (encoded by the frataxin gene). Frataxin deficiency results in impaired cellular iron metabolism and intracellular iron accumulation, reduced mitochondrial ATP production, increased free radical production, and increased oxidative stress, leading to cell death [10]. Thus, mitochondria-rich, high-energy, dependent tissues, such as the nervous system, cardiac and skeletal muscle, pancreatic beta cells, are particularly affected [11]. In the nervous system, the disease predominantly afflicts the dorsal root ganglia, sensory peripheral nerves, corticospinal and spinocerebellar tracts, and cerebellar dentate nuclei [5]. Thus, sensory, along with cerebellar ataxia, dysarthria, pyramidal weakness, absent deep tendon jerks, with extensor plantar responses are commonly seen. All these symptoms were noted in our patient as well.

MRI in Friedrich’s ataxia frequently reveals atrophy of the cervical spinal cord and medulla oblongata (sometimes associated with posterior and lateral column signal abnormality) due to widespread degeneration of the dorsal column, nucleus dorsalis, corticospinal, and spinocerebellar tracts. However, unlike other hereditary degenerative ataxias, only mild cerebellar atrophy or normal cerebellar volume is noted on neuroimaging [12,13]. MRI brain and spinal cord findings in our patient were consistent with Friedrich’s ataxia.

The triple flexion response consists of simultaneous flexion of the ankle, hip, and knee on giving a stimulus to the sole or even spontaneously. The response has been described among patients with spinal cord compression due to spinal tumors, transverse myelitis, multiple sclerosis, anterior spinal artery thrombosis, and among brain-dead patients. In a series of 38 brain-dead patients, the triple flexion response was found in only 2 out of the 38 patients (5.3%) [7]. Thus, the response is infrequently seen in actual clinical practice. Furthermore, we found only one video-recorded demonstration of the response in published literature [14]. Furthermore, the triple flexion response has never been previously video recorded in a patient with Friedrich's ataxia.

Unlike the normal withdrawal response, which is variable and inconsistent, the triple flexion response consists of stereotyped, consistent, and reproducible great toe extension along with ankle, knee, and hip flexion despite multiple stimulations. The triple flexion response has never been previously described in a patient with Friedrich’s ataxia. The descending corticospinal tract and dorsal reticulospinal tract (particularly the medullary/medial reticulospinal tracts) degeneration in Friedrich’s ataxia may cause disinhibition of spinal interneurons involved in the lower limb flexion reflex arc, producing the triple flexion response [14].

## Conclusions

To conclude, this case highlights a rare clinical sign (the triple flexion response) that has not been previously described in Friedrich’s ataxia. This is the first video documentation of the triple flexion response in Friedrich's ataxia. It is essential to correctly recognize this sign and differentiate it from a withdrawal response, as the triple flexion response highlights significant upper motor neuron dysfunction. Thus, this clinical sign may help identify profound corticospinal tract dysfunction even in advanced Friedrich's ataxia. Moreover, there is a dearth of published medical literature with actual videorecording of this sign in clinical practice. However, this is only a single case report, and further case reports demonstrating the sign in different neurological conditions (including Friedrich's and other hereditary ataxias) may bring forth other neurological disorders with the triple flexion response.

## References

[1] Buesch K, Zhang R (2022). A systematic review of disease prevalence, health-related quality of life, and economic outcomes associated with Friedreich's ataxia. Curr Med Res Opin.

[2] Campuzano V, Montermini L, Moltò MD (1996). Friedreich's ataxia: autosomal recessive disease caused by an intronic GAA triplet repeat expansion. Science.

[3] Adamec J, Rusnak F, Owen WG, Naylor S, Benson LM, Gacy AM, Isaya G (2000). Iron-dependent self-assembly of recombinant yeast frataxin: implications for Friedreich ataxia. Am J Hum Genet.

[4] González-Cabo P, Palau F (2013). Mitochondrial pathophysiology in Friedreich's ataxia. J Neurochem.

[5] Koutnikova H, Campuzano V, Foury F, Dollé P, Cazzalini O, Koenig M (1997). Studies of human, mouse and yeast homologues indicate a mitochondrial function for frataxin. Nat Genet.

[6] Jiralerspong S, Liu Y, Montermini L, Stifani S, Pandolfo M (1997). Frataxin shows developmentally regulated tissue-specific expression in the mouse embryo. Neurobiol Dis.

[7] Saposnik G, Bueri JA, Mauriño J, Saizar R, Garretto NS (2000). Spontaneous and reflex movements in brain death. Neurology.

[8] Acharya AB, Jamil RT, Dewey JJ (2025). Babinski reflex. StatPearls [Internet].

[9] Naqvi U, Margetis K, Sherman AL Muscle strength grading. StatPearls [Internet].

[10] Palau F (2001). Friedreich's ataxia and frataxin: molecular genetics, evolution and pathogenesis (review). Int J Mol Med.

[11] Campuzano V, Montermini L, Lutz Y (1997). Frataxin is reduced in Friedreich ataxia patients and is associated with mitochondrial membranes. Hum Mol Genet.

[12] Mascalchi M (2013). The cerebellum looks normal in Friedreich ataxia. AJNR Am J Neuroradiol.

[13] Pagani E, Ginestroni A, Della Nave R (2010). Assessment of brain white matter fiber bundle atrophy in patients with Friedreich ataxia. Radiology.

[14] Fujii M, Shirota S (2025). Triple flexion reflex associated with thoracic and lumbar spinal stenosis. BMJ Case Rep.

